# Draft Genome Sequence of *Microbacterium* sp. Strain HM58-2, Which Hydrolyzes Acylhydrazides

**DOI:** 10.1128/genomeA.00554-16

**Published:** 2016-06-16

**Authors:** Tomonori Akiyama, Taichiro Ishige, Yu Kanesaki, Shinsaku Ito, Ken-Ichi Oinuma, Naoki Takaya, Yasuyuki Sasaki, Shunsuke Yajima

**Affiliations:** a Department of Bioscience, Tokyo University of Agriculture, Tokyo, Japan; b NODAI Genome Research Center, Tokyo University of Agriculture, Tokyo, Japan; c Faculty of Life and Environmental Sciences, Tsukuba University, Tsukuba, Japan

## Abstract

We report the draft genome sequence of *Microbacterium* sp. strain HM58-2, which produces hydrazidase, an enzyme hydrolyzing acylhydrazides. The estimated genome size is 3.9 Mb. Genome sequence information of this strain will help to identify an assimilating mechanism of nonnatural compounds in this strain and to develop ecological applications.

## GENOME ANNOUNCEMENT

*Microbacterium* sp. strain HM58-2 has been isolated from soil to utilize an acylhydrazide, 4-hydroxybenzoic acid 1-phenyletylidene hydrazide (HBPH). This strain produces intracellular hydrazidase, which is a member of the amidase family and is considered to be a key enzyme for hydrazide catabolism (1).

Genomic DNA of *Microbacterium* sp. strain HM58-2 was extracted by using a QIAamp DNA Stool minikit (Qiagen, Valencia, CA, USA), and 3 µg DNA were fragmented to 500 bp using the Covaris S2-A system (Covaris, Woburn, MA, USA). A library for sequencing was prepared with a NEBNext DNA sample prep master mix set for Illumina (New England Biolabs, Ipswich, MA, USA) and NEXTflex PCR-free bar codes (Bioo Scientific, Austin, TX, USA). Raw reads were trimmed and *de novo* assembled using CLC Genomics Workbench version 6.0.5 (Qiagen, Valencia, CA, USA). Parameters for the trimming were as follows: ambiguous trim, yes; quality trim, yes; quality limit, 0.05; search on reversed sequence, no; save discard sequence, no; remove 5′ terminal nucleotides, no; discard short reads, no; remove 3′ terminal nucleotides, no; trim adapter list, new trim adapter list; discard long reads, no; and same broken pairs, no. Parameters for the *de novo* assembly were as follows: masking mode, no masking; mismatch cost, 3; insertion cost, 3; deletion cost, 3; length cost, 3; length fraction, 0.5; similarity fraction, 0.8; global alignment, no; and auto-detect paired distances, yes.

The draft genome of *Microbacterium* sp. strain HM58-2 was assembled into 39 contigs, with an accumulated length of 3,792,788 bp (*N*_50_ = 573,510 bp) and an average GC content of 70.1%. The longest contig was composed of 1.84 Mb, and only the five longest contigs made up a length of 3.46 Mb. The genome was annotated by the Microbial Genome Annotation Pipeline (MiGAP) version 2.19 (2), and a total of 3,565 coding sequences (CDSs), 3 rRNAs, and 45 tRNAs were predicted. Most of the genes were annotated to those of *M. testaceum* StLB037. Morohoshi et al. reported the complete genome sequence of *M. testaceum* StLB037 comprising 3.98 Mb with 3,532 CDSs and an average GC content of 70.28% (3). The draft genome sequence of strain HM52-8 was, therefore, expected to cover almost the full sequence of the bacterium.

Since the expression of the hydrazidase gene is under catabolite repression, where it is induced by substrates, such as HBPH, as sole carbon source and repressed by the existence of glucose (1). Therefore, the genome sequence of strain HM58-2 will facilitate the understanding of this bacterium’s role in the catabolic mechanism of acylhydrazides.

### Nucleotide sequence accession numbers.

This whole-genome shotgun project has been deposited at DDBJ/EMBL/GenBank under the accession numbers BDCY01000001 to BDCY01000039.
